# Pressure-Sensitive Nano-Sheet for Optical Pressure Measurement

**DOI:** 10.3390/s21217168

**Published:** 2021-10-28

**Authors:** Yu Matsuda, Riki Orimo, Yusaku Abe, Yuri Hiraiwa, Yosuke Okamura, Yuta Sunami

**Affiliations:** 1 Department of Modern Mechanical Engineering, Waseda University, 3-4-1 Ookubo, Shinjuku-ku, Tokyo 169-8555, Japan; orimori987@fuji.waseda.jp (R.O.); ya-jupiter0309@toki.waseda.jp (Y.A.); 2 Japan Science and Technology Agency (JST), PRESTO, 4-1-8 Honcho, Kawaguchi 332-0012, Saitama, Japan; 3 Department of Mechanical Engineering, Tokai University, 4-1-1 Kitakaname, Hiratsuka 259-1292, Kanagawa, Japan; yurihiraiwa408@gmail.com (Y.H.); sunami@tokai-u.jp (Y.S.); 4 Department of Applied Chemistry, School of Engineering, Tokai University, 4-1-1 Kitakaname, Hiratsuka 259-1292, Kanagawa, Japan; y.okamura@tokai-u.jp; 5 Micro/Nano Technology Center, Tokai University, 4-1-1 Kitakaname, Hiratsuka 259-1292, Kanagawa, Japan; 6 Research Institute of Science and Technology, Tokai University, 4-1-1 Kitakaname, Hiratsuka 259-1292, Kanagawa, Japan

**Keywords:** pressure-sensitive paint, nano-sheet, pressure-sensitive nano-sheet, pressure measurement

## Abstract

Pressure-Sensitive Paint (PSP) is a powerful measurement technique to obtain pressure distribution on a model of interest by measuring the emission intensity of the PSP coating with a camera. Since a PSP coating is prepared by applying a solution containing an organic solvent, generally, by sprayer, the properties such as the pressure- and the temperature-sensitivity depends on the skill of the person applying it. This fabrication process is one of the barriers to use of the PSP technique because of the legal restrictions on the use of organic solvents. Thus, a sticker-like PSP coating is useful because it does not require the use of organic solvent and the applying skill. In this study, we have fabricated freestanding Pressure-Sensitive Nano-Sheet (PSNS) by a sacrificial layer process using a spin-coating method. We employed Pt(II) meso-tetra(pentafluorophenyl)porphine (PtTFPP) as a pressure-sensitive dye and poly(1-trimethylsilyl-propyne) (PTMSP) and poly(L-lactic acid) (PLLA) as a polymer binder; thus, the PSNS samples based on PTMSP and PLLA were prepared. The pressure- and the temperature-sensitivity, the lifetime of the luminescence, and the quantum yield of the fabricated PSNS have been investigated. The pressure-sensitivity of PTMSP-based PSNS is higher than that of PLLA-based PSNS. Conversely, the quantum yield of PLLA-based PSNS is higher than that of PTMSP-based PSNS.

## 1. Introduction

As a non-intrusive pressure measurement technique for fluid mechanics, pressure-sensitive paint (PSP) [1,2,3,4] has drawn much attention. For example, the PSP method has been applied to wind tunnel testing [5,6,7,8,9,10,11], surface pressure measurement on rotating objects [12,13,14,15], low density gas flow measurements [16,17,18], oxygen concentration distribution measurements [19,20], and micro-scale gas flow measurements [21,22,23,24]. The pressure distribution on a surface to which a PSP coating is applied can be measured by detecting the variation of the luminescence intensity emitted from the pressure-sensitive dyes in the PSP coating. In general, a PSP coating consists of pressure-sensitive dyes contained in an oxygen-permeable polymer binder. A PSP coating is prepared by dissolving pressure-sensitive dyes and a polymer binder in an organic solvent (e.g., toluene, dichloromethane, chloroform, and tetrahydrofuran), and the resulting solution is applied to a model surface by a sprayer or an airbrush. The properties such as the pressure- and the temperature-sensitivity depend on the skill of the person applying it. Moreover, this fabrication process is one of the barriers to use of the PSP technique because of the legal restrictions on the use of organic solvents. A PSP coating cannot be applied to a plastic surface without resistance to organic solvents. Thus, a sticker-like PSP coating is useful because it does not require the use of organic solvent and the applying skill.

In recent years, there are many studies on the fabrication methods of nano-sheets for wearable sensors and in vitro cellular studies [25,26,27,28,29,30]. In general, nano-sheets have unique properties such as high transparency, high flexibility, noncovalent adhesion, and excellent electrical and thermal properties due to a large size-aspect ratio [26,29]. The properties such as high flexibility and noncovalent adhesion are also suitable for PSP. The authors had developed a nano-sheet PSP, called as pressure-sensitive molecular film (PSMF) [31,32], based on the Langmuir–Blodgett (LB) method [33,34]. However, the fabrication of PSMF based on the LB method is time consuming, and a special apparatus called “LB trough” is needed. Since the LB method is only applicable for chemically modified flat surfaces, PSMS cannot be peeled off from the substrate and be used as a sticker-like sensor. Sakamura et al. [35] proposed a monolayer PSP based on a self-assembled monolayer (SAM) process. Pt(II)-porphyrin is synthesized and covalently attached to the surface of indium tin oxide (ITO) glass plates by a SAM process. Their SAM-PSP also cannot be peeled off from the substrate (ITO glass). Then, the existing nano-sheet PSP coatings are not suitable for a sticker-like PSP coating.

In this study, we have fabricated freestanding pressure-sensitive nano-sheet (PSNS) using a sacrificial layer process. The fabricated PSNS can be peeled off from the substrate and sticked to another surface. In this paper, we have investigated the basic properties of PSNF fabricated by a spin-coating method [26] before mass-production by a roll-to-roll process. The pressure- and the temperature-sensitivity, the lifetime of the luminescence, and the quantum yield of the fabricated PSNS have been investigated in this study.

## 2. Basic Principles of PSNS

The PSNS consists of pressure-sensitive dyes and polymer binder. The basic principle of PSNS is the same as ordinary PSPs. The pressure-sensitive dyes are photo-excited by absorption of photons from an illumination light source. Then, the excited dyes emit the phosphorescence. Here, the quantum yield is defined as the fraction of absorbed photons that produces phosphorescence [2,36]. Then, the higher quantum yield means the higher emission intensity. In the actual pressure measurement, the brighter PSP leads to higher signal-to-noise ratio (SNR) measurement, and PSP with high quantum yield is desirable. The lifetime is defined by the average time that the dye spends in the excited state before returning to the ground state [36]. The interaction between the dyes and oxygen molecules leads to decrease in the emission intensity during the lifetime of the phosphorescence. This process is known as oxygen quenching. Then, the emission intensity depends on pressure near the surface [1,2,37], and the relationship between the emission intensity and pressure is represented by the Stern–Volmer equation as follows:

(1) $$\frac{I_{\mathrm{ref}}}{I}=A+B\frac{p}{p_{\mathrm{ref}}}$$

where I and p are the emission intensity and pressure, respectively. The emission intensity described as $I_{\mathrm{ref}}$ is the intensity at a reference pressure $p_{\mathrm{ref}}$. In this study, an atmospheric pressure is used as the reference pressure $p_{\mathrm{ref}}$. The coefficients A and B are called the Stern–Volmer coefficients and are determined by a calibration test. The pressure-sensitivity [1] is defined as follows:

(2) $$S_{p}=\frac{\partial }{\partial p}\frac{I_{\mathrm{ref}}}{I}=\frac{B}{p_{\mathrm{ref}}}$$

The emission intensity and the Stern–Volmer coefficient usually depend on temperature; thus, the temperature was kept at a constant temperature of 25 °C during the pressure-sensitivity test in this study.

The relation between the emission intensity and temperature can be approximately written in the Arrhenius form [1,4]. However, some PSPs deviates from the Arrhenius form. In this study, the relation of our PSNS was well expressed by the following simple linear equation:

(3) $$\frac{I}{I_{\mathrm{ref}}}=C+D\frac{T}{T_{\mathrm{ref}}}$$

where T is the temperature. The intensity described as $I_{\mathrm{ref}}$ is the intensity at a reference temperature $T_{\mathrm{ref}}=25$ °C. The coefficients C and D are determined by a calibration test. It is noted that $I_{\mathrm{ref}}$ is the denominator unlike the Stern–Volmer equation (Equation (1)). The temperature-sensitivity is also defined by the following equation [11,38,39,40]:

(4) $$S_{T}=\frac{\partial }{\partial T}\frac{I}{I_{\mathrm{ref}}}=\frac{D}{T_{\mathrm{ref}}}$$

## 3. Materials and Methods

### 3.1. Fabrication of PSNS

We prepared two kinds of PSNSs with different polymers: poly(1-trimethylsilyl-propyne) (PTMSP, NOF Corporation, Tokyo, Japan) and poly(L-lactic acid) (PLLA, Funakoshi, Japan). PTMSP is known as a glassy polymer having high gas permeability [41,42], and is usually used as a binder of PSP. In general, however, PTMSP is not used for the fabrication of nano-sheet. While PLLA is one of polymer materials used to fabricate a nano-sheet by a spin-coating method [26,43,44] and a roll-to-roll process [45], PLLA is not used as a binder of PSP. As a pressure-sensitive dye, Pt(II) meso-tetra(pentafluorophenyl)porphine (PtTFPP, Porphyrin-Laboratories, Scharbeutz, Germany) was employed for both PSNS samples. PtTFPP is widely used as pressure-sensitive dye due to high pressure-sensitivity and optical stability [1,2,38,39,46,47].

**Figure 1:**
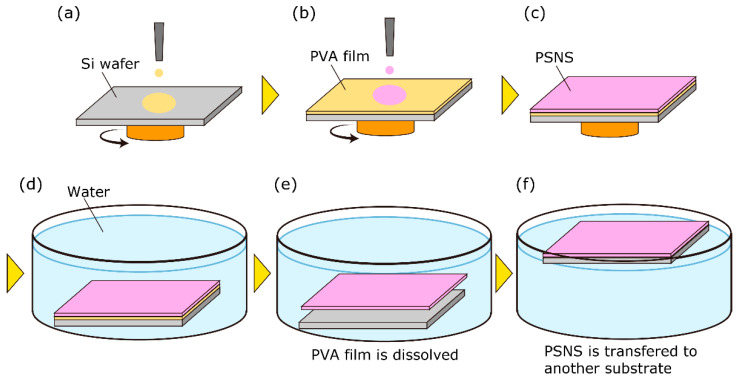
Schematic of PSNS fabrication process based on a sacrificial layer process using a spin-coating method. (**a**) PVA solution is spin-coated on Si wafer. (**b**) PSNS is spin-coated on the PVA film. (**c**) fabricated PSNS and PVA film (**d**) Obtaied film is immersed in water. (**e**) PVA film is dissolved and free standing PSNS is obtained. (**f**) PSNS is transferred to another substrate.

**Figure 2:**
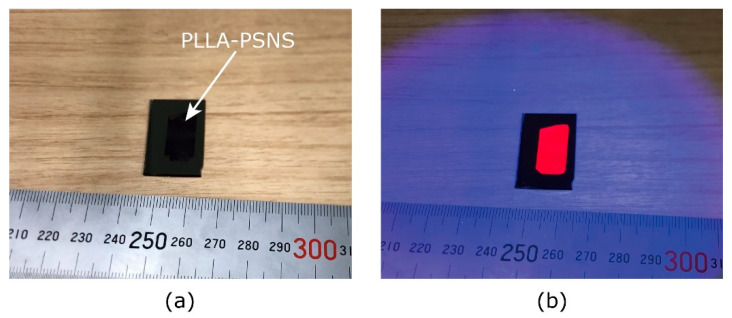
Typical example of PLLA-PSNS transferred on silicon wafer. (**a**) PLLA-PSNS image without illumination. (**b**) PLLA-PSNS image with illuminationation.

Figure 1 shows the PSNS fabrication process used in this study. PSNS was fabricated by a spin-coating method as follows: First, the solutions of PtTFPP and polymer were prepared. PtTFPP and PTMSP/PLLA were dissolved in an organic solvent (toluene for PTMSP and chloroform for PLLA) with the concentrations of 1.0 mg/mL and 10.0 mg/mL, respectively. We prepared the PtTFPP and the PTMSP/PLLA solution separately. Each solution was stirred 400 rpm for 5 h, and the PtTFPP and the PTMST/PLLA solution was mixed and stirred again 400 rpm for 5 h. These recipes follow the recipes of general PSPs [4,19]. The aqueous solution of poly(vinyl alcohol) (PVA, FUJIFILM Wako Chemicals Corporation, Osaka, Japan) with the concentration of 20 mg/mL was also prepared. Second, the PVA solution (about 0.1 mL) was coated on a silicon wafer surface by a spin-coater (MS-A 150, Mikasa, Tokyo, Japan) with 1000 rpm for 100 s. The fabricated PVA film works as a water-soluble sacrificial film [43]. Third, using the PtTFPP and polymer solution, we fabricated the PSNS film on the PVA film by the spin coater with 1000 rpm for 100 s. Last, by dissolving the PVA sacrificial film in distilled water, we obtained freestanding PSNS film and transferred it on another substrate (aluminum, Teflon, and another silicon wafer plates are examined in this study). All processes were carried out at room temperature. The PTMSP and the PLLA-based PSNS samples will be referred as PTMSP-PSNS and PLLA-PSNS, respectively. Figure 2 shows the typical example of the PLLA-PSNS transferred from a silicon wafer to another one. The images were taken by iPhone 8.

The thickness of the fabricated PSNS samples were measured by a stylus profilometer (DektakXT, Bruker, Billerica, MA, USA). The thickness of the PTMSP-PSNS sample and that of the PLLA-PSNS were 22 nm and 91 nm, respectively. The thickness of our PSNS is much thinner than that of conventional sprayed PSP coating of $O\left(1\right)$ μm [4]. The difference in the thickness between PTMSP-PSNS and PLLA-PSNS was considered as the result of the difference in the viscosity between the PTMSP and the PLLA organic solution. 

### 3.2. Experimental Setup

The pressure- and the temperature-sensitivity of the fabricated PSNSs were investigated using a calibration chamber. The calibration chamber was similar to our previous studies [11,48]. The PSNS samples were placed in the calibration chamber, and the pressure in it was monitored and controlled by a pressure controller (PACE5000, Baker Hughes, Houston, TX, USA). The temperature in the chamber was measured by a thermistor (B57861S0103F045, TDK Electronics, Tokyo, Japan) and was monitored and controlled by a Peltier device (TEC1-12708, Kaito Denshi, Hasuda, Japan) and a temperature controller (TDC-1020a, Cell System, Yokohama, Japan). For the pressure-sensitivity test, we controlled the pressure in the chamber in the range of 50 to 110 kPa and kept the temperature in it at 25 °C. For the temperature-sensitivity test, we controlled the temperature in the chamber in the range from 20 to 45 °C and kept the pressure at an atmospheric pressure. The PSNS was illuminated by an LED device, the central wavelength of which was 395 nm (LED294-395, Hamamatsu, Japan). The emission from the PSNS was captured by a CCD camera (PCO.1600, PCO AG, Kelheim, Germany) with a band-pass filter of 630 ± 30 nm (Asashi Spectra, Tokyo, Japan).

The quantum yields of the fabricated PSNSs were measured by an absolute quantum yield spectrometer (Quantaurus-QY, C11347-01, Hamamatsu Photonics, Hamamatsu, Japan). The lifetimes of the phosphorescence emitted from the PSNS samples were measured by a lifetime spectrometer (Quantaurus-Tau, C11367-31, Hamamatsu Photonics, Hamamatsu, Japan). In the lifetime spectrometer, the lifetime is obtained by fitting the data with triple exponential functions.

## 4. Results and Discussion

**Figure 3:**
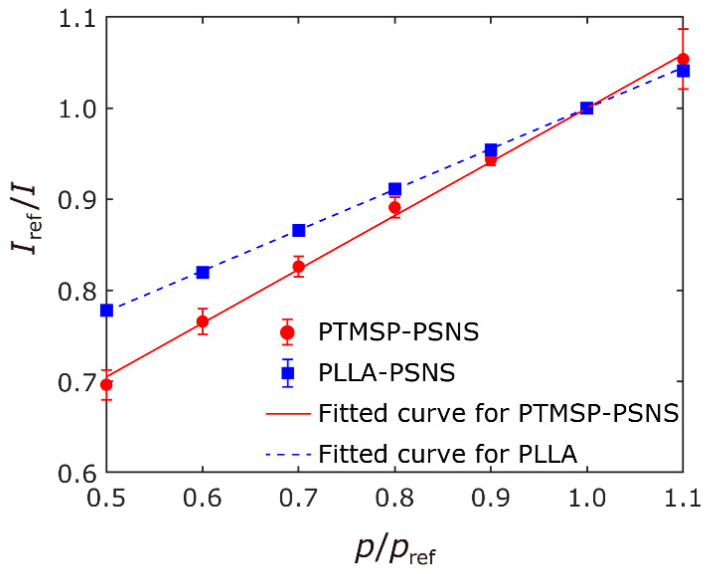
Stern–Volmer plots for PTMSP-PSNS and PLLA-PSNS samples.

**Figure 4:**
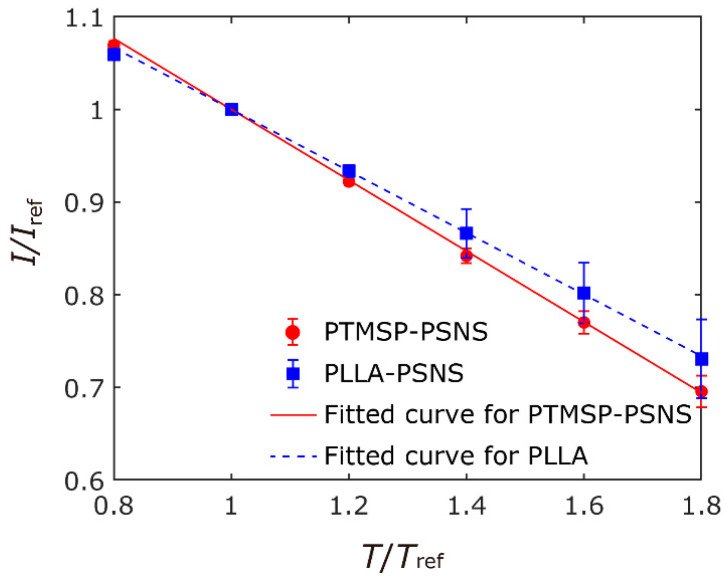
Result of temperature calibration tests for PTMSP-PSNS and PLLA-PSNS samples.

The results of the pressure- and the temperature-sensitivity test were shown in Figure 3 and Figure 4, respectively. The error bars indicate the standard deviation of the intensity ratio on the sample coupons. As shown in Figure 3, the pressure sensitivity of PTMSP-PSNS ($S_{p}=0.59\%/\mathrm{kPa}$) was higher than that of PLLA-PSNS ($S_{p}=0.45\%/\mathrm{kPa}$). Though the pressure-sensitivity of PTMSP-PSNS is smaller than that of conventional sprayed PSP with the same components ($S_{p}=0.72\%/\mathrm{kPa}$ is reported for sprayed PtTFPP/PTMSP coating in [4,41]), there are many existing PSPs with similar pressure-sensitivities [1], and the fabricated PSNS works as pressure sensor. The temperature-sensitivities of PTMSP-PSNS and PLLA-PSNS were $S_{T}=-1.52\%/℃$ and −1.32%/℃, respectively. The temperature-sensitivity of our PTMSP-PSNS is higher than that of sprayed PtTFPP/PTMSP coating of $S_{T}=-0.29\%/℃$ [4,41]. This temperature-sensitivity is similar to sprayed PtTFPP/Poly(tBS) coating of $S_{T}=-1.7\%/℃$ [4].

We measured the quantum yields of PTMSP-PSNS and PTMSP-PSNS on a Teflon and an aluminum plate at an atmospheric pressure and the temperature of 24 °C. The results are shown in Table 1. The quantum yields of samples on a Teflon plate were about 2 times higher than those on an aluminum plate. These results indicated that the diffuse reflection of the emission light on a white Teflon plate increased the emission rate. Moreover, the quantum yield of PLLA-PSNS was 6.5 to 8.4 times higher than that of PTMSP-PSNS.

**Figure 5:**
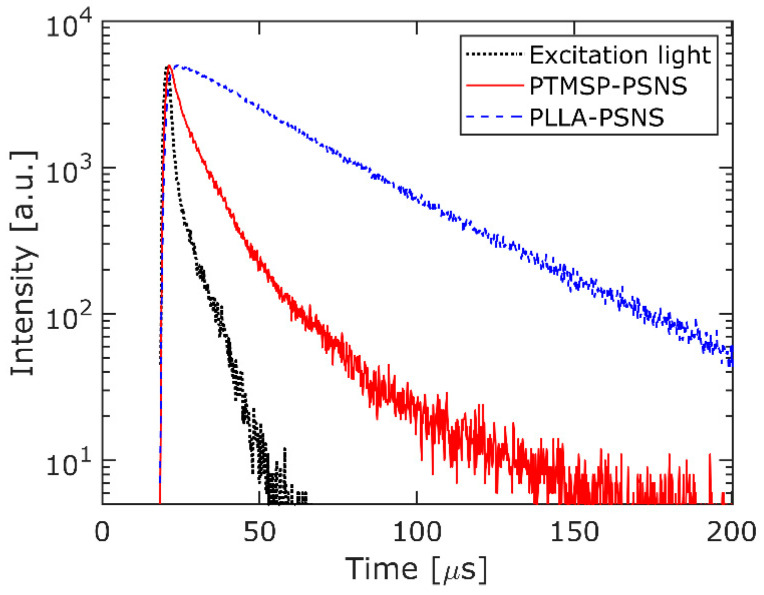
Phosphoresence decay curves for PTMSP-PSNS and PLLA-PSNS.

In contrast to the quantum efficiency, the difference in the lifetimes between the PSNS on the Teflon and that on the aluminum was small (within 10%). Then, as a typical example, Figure 5 shows that the phosphorescence decay curves of the PTMSP-PSNS and the PLLA-PSNS samples on the Teflon plate at an atmospheric pressure and the temperature of 24 °C measured by the lifetime spectrometer. From Figure 5, the lifetimes of the PTMSP-PSNS and the PLLA-PSNS samples are obtained as 8.5 μs and 32.8 μs, respectively. The lifetime of PTMSP-PSNS of 8.5 μs was similar to that reported in the literature [14,18]. Interestingly, the lifetime of the PLLA-PSNS sample was 3.9 times longer than those of the PTMSP-PSNS samples and the conventional sprayed PtTFPP-based PSPs [14,18]. In general, the lifetime is independent of the number of pressure-sensitive dyes in the layer and depends on the concentrations of quencher (oxygen molecule) and the pressure-sensitive dyes (due to concentration quenching). In our condition, the concentrations of the pressure-sensitive dye were similar to each other. This result indicates that the oxygen concentration in the PLLA-PSNS layer is lower than that in the PTMSP-PSNS layer. It is known that the oxygen permeability coefficient and the diffusion coefficient of a PTMS layer are about $7\times 10^{-7}\;{\mathrm{cm}}^{3}\left(\mathrm{STP}\right)\mathrm{cm}/{\mathrm{cm}}^{2}\;s\;\mathrm{cm}\;\mathrm{Hg}$ and $5\times 10^{-5}\;{\mathrm{cm}}^{2}/s$, respectively, [3,49,50]. On the other hand, the oxygen permeability coefficient and the diffusion coefficient of a PLA layer are about $8\times 10^{-10}\;{\mathrm{cm}}^{3}\left(\mathrm{STP}\right)\mathrm{cm}/{\mathrm{cm}}^{2}\;s\;\mathrm{cm}\;\mathrm{Hg}$ and $4\times 10^{-8}\;{\mathrm{cm}}^{2}/s$, respectively. The differences in quantum yield and lifetime of PTMSP-PSNS and PLLA-PSNS may be attributed to the differences in the oxygen permeability and the diffusion coefficient. The low oxygen permeability and diffusivity prevent the oxygen quenching in the PLLA-PSNS layer, increasing the quantum yield, and extending the lifetime. On the other hand, the high oxygen permeability and diffusivity of PTMSP-PSNS lead to high pressure sensitivity, low quantum yield, and short lifetime of the phosphorescence. As a result, PLLA-PSNS is suitable for pressure measurement from the viewpoint of the high SNR measurement. On the other hand, PTMSP-PSNS is suitable from the viewpoint of the pressure-sensitivity.

## 5. Conclusions

We fabricated two kinds of pressure-sensitive nano-sheet (PSNS) based on poly(1-trimethylsilyl-propyne) (PTMSP) and poly(L-lactic acid) (PLLA) as a polymer binder and called them PTMSP-PSNS and PLLA-PSNS, respectively. Pt(II) meso-tetra(pentafluorophenyl)porphine (PtTFPP) was used as a pressure-sensitive dye. The fabricated PSNS can be peeled off from the substrate by dissolving a sacrificial film, poly(vinyl alcohol) (PVA) in this study, and can be transferred to another substrate. Though both PTMSP-PSNS and PLLA-PSNS were fabricated by a spin-coater with the same rotational speed, the thickness of PTMSP-PSNS and PLLA-PSNS were 22 nm and 91 nm, respectively. This difference in the thickness was considered as the result of the viscosity between the PTMSP and the PLLA organic solution. We examined the pressure-sensitivity of both PSNSs. As a result, pressure-sensitivity of PTMSP-PSNS (0.59 %/kPa) is higher than that of PLLA-PSNS (0.45 %/kPa). The pressure-sensitivity of the fabricated PSNS is similar to those of conventional PSPs. The quantum yields and the lifetimes of the PSNS samples were also measured. The quantum yield of PLLA-PSNS is much higher than that of PTMSP-PSNS and the lifetime of PLLA-PSNS is much longer than that of PTMSP-PSNS. These results indicate that the oxygen quenching in PLLA-PSNS is prevented by the low oxygen permeability of PLLA. Moreover, it is clarified that a white substrate improves the quantum yield of PSNS. 

In this study, we investigated the basic properties of PSNS. As future work, for example, the spatial uniformity of PSNS should be investigated for micro-scale flow applications, and a roll-to-roll fabrication method providing larger PSNS for the application of wind tunnel testing should be studied. 

## Figures and Tables

**Table 1:** Quantum yields of PTMSP-PSNS and PLLA-PSNS.

	Quantum Yield (Teflon Plate)	Quantum Yield (Aluminum Plate)
PTMSP-PSNS	0.014	0.007
PLLA-PSMS	0.091	0.059

## Data Availability

Data sharing not applicable.
